# Effects of Whey Protein Supplementation on Inflammatory Marker Concentrations in Older Adults

**DOI:** 10.3390/nu15184081

**Published:** 2023-09-21

**Authors:** Samuel Adler, Wyatt Olsen, Bryna Rackerby, Rachel Spencer, David C. Dallas

**Affiliations:** 1 Department of Food Science & Technology, Oregon State University, Corvallis, OR 97331, USAwyatt.olsen@oregonstate.edu (W.O.); bryna.rackerby@oregonstate.edu (B.R.); 2 Nutrition Program, College of Health, Oregon State University, Corvallis, OR 97331, USA; spenrach@oregonstate.edu

**Keywords:** dairy, inflammaging, immunomodulation, chronic inflammation

## Abstract

Although whey protein isolate (WPI) has been shown to be immunomodulatory, its ability to modulate production of a broad array of inflammatory markers has not previously been investigated in healthy adults. We investigated the effects of daily supplementation with 35 g of WPI for 3 weeks on inflammatory marker concentrations in the blood serum and feces of 14 older adult subjects (mean age: 59). Serum was analyzed using a multiplex assay to quantify the cytokines IFN-γ, IL-1β, IL-1RA, IL-2, IL-3, IL-4, IL-5, IL-6, IL-7, IL-8, IL-9, IL-10, IL-12p70, IL-13, IL-17A and TNF-α. Fecal samples were analyzed using an ELISA for the inflammatory markers calprotectin and lactoferrin. Our results yielded high inter-subject variability and a significant proportion of cytokine concentrations that were below our method’s limit of quantification. We observed decreases in serum IL-12p70 in the washout phase compared with baseline, as well as the washout stage for fecal lactoferrin relative to the intervention stage. Serum IL-13 was also significantly reduced during the intervention and washout stages. Our data suggest that whey protein supplementation did not significantly alter most inflammatory markers measured but can alter concentrations of some inflammatory markers in healthy older adults. However, our study power of 35% suggests the number of participants was too low to draw strong conclusions from our data.

## 1. Introduction

At least one in 20 people in the western world are thought to have a chronic inflammatory disease [1]. Chronic inflammation is associated with inflammatory bowel disease (IBD) and other illnesses that can severely decrease quality of life. Even low-grade systemic inflammation may contribute to disease: it has been associated with arteriosclerosis, neurodegenerative disease and type 2 diabetes [2,3]. However, not all chronic inflammation manifests as a disease state. Low-grade inflammation becomes more common as people age and is termed “inflammaging” [4]. The cause of inflammaging is not well understood. It is thought to begin in adulthood for most people and can increase risk of morbidity and mortality [5].

Chronic inflammation is mediated by cytokines, small proteins that promote or regulate the state of inflammation. High levels of the cytokines interleukin (IL)-1, IL-8, IL-10, IL-13, TNF-α and especially IL-6 are present in adults with inflammaging [4,5,6] and are linked to the onset of illnesses such as type 2 diabetes mellitus, osteoporosis, Alzheimer’s disease, rheumatoid arthritis and coronary heart disease [7,8]. Potentially, changes in diet could alter inflammatory protein profiles, which could lessen inflammation-associated disease states.

Whey proteins could be a dietary modulator of inflammation. WPI, derived from cheesemaking, is composed of several subtypes of proteins and peptides. Whey protein is made up of hundreds of unique proteins [7]. Among these, the whey proteins lactoferrin and α-lactalbumin and the κ-casein-derived peptide glycomacropeptide (GMP) have various bioactivities, including immunomodulatory properties [8,9,10]. Moreover, as whey protein is digested, it releases an array of peptides [11]. Many bovine milk whey protein-derived peptides have bioactivities, including some with immunomodulatory function [12].

Individual whey protein components have shown promise in reducing inflammation. For example, a review by Córdova-Dávalos et al. indicated that GMP can have either anti-inflammatory or pro-inflammatory properties in cells and animal models, with most studies reporting anti-inflammatory effects [13]. The predominant mechanisms ascribed to these anti-inflammatory effects of GMP were a down-regulation of the MAPK and NF-κB inflammatory signaling pathways [13]. Moreover, Feeney et al. demonstrated that GMP can limit intestinal epithelial cell barrier dysfunction and decrease the adhesion of pathogenic *Escherichia coli* strains, suggesting its potential to prevent interactions (e.g., lipopolysaccharide (LPS) binding to toll-like receptor 4 (TLR4)) that would lead to gut inflammation [14]. A review by Baveye et al. indicated that lactoferrin modulates immune cell cytokine production in vitro and in mice via both inhibition of inflammatory cytokines associated with inflammaging, such as TNF-α and IL-1β, and promotion of anti-inflammatory IL-10 [15,16].

In addition to these studies of individual whey proteins, the impact of WPI supplementation as a whole has been studied in human subjects [17]. At least 18 research experiments on WPI in humans have examined the ability of WPI to modulate immune function in older adults, although only two have investigated healthy older adults [17]. A meta-analysis by Prokopidis et al. found no overall change in TNF-α, but a significant overall reduction in IL-6 with whey protein supplementation in studies with an average participant age > 50 [17]. However, as mentioned in Prokopidis et al., further studies on the effects of whey proteins on inflammatory status in older adults are needed. Moreover, past studies in healthy older adults have examined relatively few immune markers, and few have examined the gut-specific immune effects.

Therefore, the goal for our study was to examine how daily WPI supplementation in healthy older adults alters a wide array of systemic and gut-specific inflammatory markers (i.e., blood cytokines and fecal calprotectin and lactoferrin).

## 2. Materials and Methods

Subject recruitment and selection. Informational flyers about the study were posted in public locations in Benton and Linn County, Oregon, USA. Twenty-two potential participants responded to the contact information on the flier and were screened for inclusion criteria: (1) between 50 and 70 years old, (2) have daily bowel movements, (3) no lactose intolerance or dairy allergy/intolerance, (4) no history of major gastrointestinal illness/surgery, (5) no history of laxative or antacid use, and (6) no antibiotics taken in the month prior to sample collection. A total of 14 subjects (9 female and 5 male, mean age: 59, age range: 51–70) were selected for participation in the trial.

Whey protein supplement. Upon enrollment in the study, participants were given 21 pre-portioned servings (59 g each) of a chocolate-flavored, sucrose-sweetened mix containing 35 g of WPI Provon 290 (Glanbia Nutritional, Twin Falls, ID, USA) (Table 1). Participants were instructed to rehydrate this protein powder with 8 oz (237 mL) water and consume the full volume daily; most reported doing so in the morning.

*Feeding trial design and sample collection.* The trial period was a total of 7 weeks. For the first week (week 1: baseline), subjects were asked to make no changes to their diet. For each of the following 3 weeks (weeks 2–4: intervention), subjects were instructed to consume a single serving of whey protein supplement (described above) daily. For the final 3 weeks (weeks 5–7: washout), subjects returned to their normal diets. Participants were instructed to collect a stool sample using the EasySampler Stool Collection Kit (ALPCO, Salem, NH, USA) on a Tuesday or Wednesday during each of the 7 weeks of the study depending on sampling schedule. They were then instructed to deliver their stool at ambient temperature to the Dallas lab at Oregon State University within 1 h of collection. Upon receipt by the laboratory, about 10 g of the stool samples were transferred into stool collection tubes (Norgen, Thorold, ON, Canada) and stored at −80 °C for later analysis within 3 h of collection. On stool sample collection days, participants were also instructed to go to the Samaritan Health Services laboratory for blood sample collection. Upon arrival at the laboratory, a phlebotomist collected 10 mL of blood into heparin-coated tubes, which were immediately placed on ice. Within 30 min, blood samples were centrifuged at 750× *g* for 15 min to remove blood cells. The supernatant was transferred to 15 mL conical centrifuge tubes (Corning, Corning, NY, USA) and frozen at −80 °C. Prior to further analysis, blood serum was thawed by holding at −20 °C for 24 h followed by 4 °C for 6–8 h on ice. Thawed blood serum was separated into 0.5 mL aliquots in 1.5 mL plastic microcentrifuge tubes and returned to storage at −80 °C for later analysis via multiplex.

Fecal sample analysis for fecal calprotectin. Calprotectin is an antimicrobial protein secreted by neutrophils in the gut, and higher levels are associated with IBD [18]. Frozen fecal samples (10 g) were thawed at ambient temperature for 6 h, and a total of 15 mg or 15 μL of stool (depending on stool consistency) was collected from three distinct locations within each sample via the ALPCO easy stool extraction kit (ALPCO, Salem, NH, USA). Upon collection, the samples were added to the supplied extraction buffer (1.5 mL) and underwent orbital shaking at 1000 rpm for 30 min to solubilize the sample. Fecal extracts were stored at 4 °C and analyzed within 3 days by ELISA at 2500× dilution (ALPCO Fecal Calprotectin Absorbance-Based ELISA Kit) in duplicate on a Spectramax M2 spectrophotometer (Molecular Devices, San Jose, CA, USA). Three plates were read at 450 nm against a reference wavelength of 620 nm that was subtracted from each reading in compliance with the protocol. All three plates had a standard curve R^2^ > 0.973. Two manufacturer-provided positive controls were included on each plate and were within the accepted range of the assay.

Fecal sample analysis for fecal lactoferrin. Lactoferrin is an antimicrobial protein secreted by neutrophils in the gut, and higher levels are associated with IBD [18]. Fecal extracts were prepared in an identical way to the calprotectin extracts, except the IDK Extract^®^ universal stool extraction buffer (Immundiagnostik AG, Bensheim, Germany) was used instead of the ALPCO easy stool extraction kit. Samples were analyzed with the Faecal Lactoferrin ELISA (Immundiagnostik) at 1000× dilution in duplicate on a Spectramax M2 spectrophotometer. Three plates were again read at 450 nm against a reference wavelength of 620 nm that was subtracted from each reading in compliance with the protocol. All three plates had a standard curve R^2^ > 0.938. Two manufacturer-provided positive controls were included on each plate and were within the accepted range of the assay.

Blood serum analysis for cytokines. Cytokine concentrations in blood serum samples were determined following the protocol outlined in the Milliplex^®^ Human Cytokine/Chemokine/Growth Factor Panel A—Immunology Multiplex Assay (Millipore Sigma, Burlington, MA, USA). The custom panel was designed to quantify 16 targets: IFN-γ, IL-1β, IL-1RA, IL-2, IL-3, IL-4, IL-5, IL-6, IL-7, IL-8, IL-9, IL-10, IL-12p70, IL-13, IL-17A and TNF-α. Frozen blood serum samples were thawed on ice and centrifuged at 1000× *g* for 10 min at 4 °C. A 25 μL volume of serum supernatant was added without dilution to each well on a 96-well plate and mixed with 25 μL of the customized mixture of magnetic beads. Samples were incubated with standards and positive controls on a shaker-plate at 4 °C for 12–16 h. Incubated plates were loaded onto the Luminex 200 (Luminex Corporation, Austin, TX, USA) and analyzed using the xPONENT^®^ data acquisition software (Version 4.3.309.1). The xPONENT software converts mean fluorescent intensity (MFI) values to analyte concentration based on the integrated standard curve. The range of quantification and limit of quantification (LOQ) are shown in Table 2. Calibration curve R^2^s are reported in Supplementary Table S1.

Statistical analysis. Concentrations of cytokines and inflammatory markers were analyzed for differences between study stages (baseline, treatment and washout) using a repeated measures ANOVA in JMP Pro software Version 17.0.0 (623769) with significance designated as a *p*-value < 0.05 (stage model). An additional repeated measures ANOVA was conducted in which the groups were the 7 weeks of the study, as opposed to the three stages (week model). In these ANOVAs, a mixed model ANOVA was run with the effect of study participant being assigned as a random variable and stage or week as a model effect.

Study power was calculated in G*Power 3.1 in an “ANOVA: repeated measures, between factors” using the following parameters in post hoc analysis: effect size = 0.2, α = 0.05, sample size = 14, number of groups = 1, number of measurements = 3 and correlation among repeated measures = 0.55. Ideal sample size was computed using an a priori analysis with study power equal to the power output of the post hoc analysis. Based on Cohen’s suggestion, an effect size of 0.2 was selected for a small effect [19].

Two extreme outliers were removed: one participant’s IL-12p70 and TNF-α week 1 (baseline) data points, as they were more than two orders of magnitude greater than the second highest value among all participants in the study, and their inclusion significantly altered ANOVA data interpretation.

## 3. Results

The average concentration and standard error of serum cytokines and fecal inflammatory markers were assessed for each stage of the study (Table 3).

Change in inflammatory marker concentrations measured by ANOVA. The effect of WPI consumption on serum cytokine and fecal inflammatory biomarkers was assessed via repeated measures ANOVA, substituting the LOQ for values below the LOQ. Tukey’s honest significant difference (HSD) test was conducted in post hoc analysis for inflammatory markers in which a *p* < 0.05 was observed in order to determine what significant changes occurred. In the stage model, significant changes were observed in serum concentrations of IL-12p70 (*p* = 0.01), IL-13 (*p* = 0.0005) and fecal lactoferrin (*p*=0.02) (Table 4). Only IL-13 changed significantly in the week model (*p* = 0.007) (Table 4). No other significant ANOVA main effects were observed. No IL-3 was quantified. All repeated measures ANOVAs for each inflammatory marker reported a significant effect from study participants (*p* < 0.0219), suggesting that concentrations vary significantly depending on the subject.

**Figure 1:**
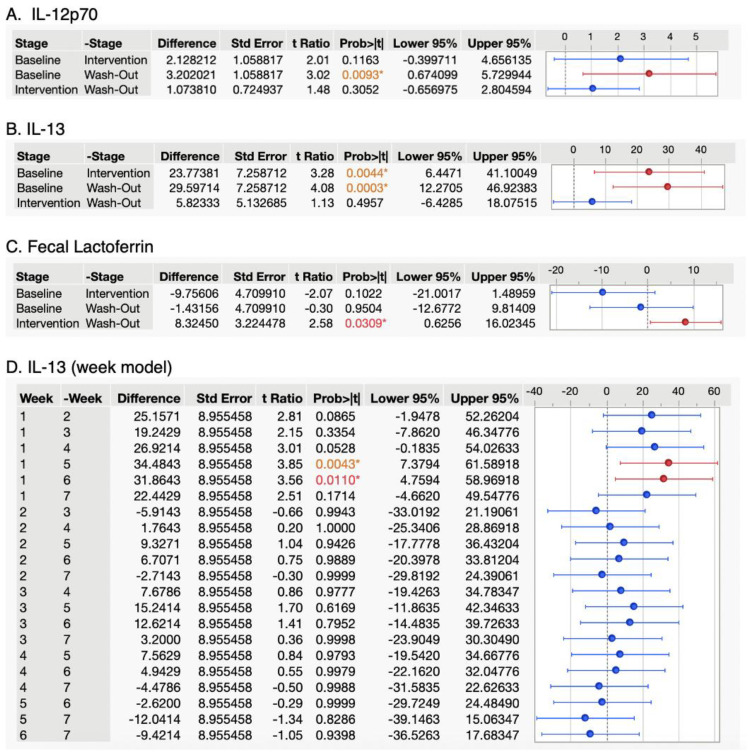
Tukey’s HSD test for inflammatory markers with significant ANOVA *p*-values. Change in expression of inflammatory markers for which at least one stage or week was significantly different from any other. Horizontal error bars denote the concentration differences that represent the 95% confidence interval of the difference between the two stages represented in the HSD test. A * symbol indicates a statistically significant *p*-value, red text indicates a *p*-value less than 0.05, orange text indicates a *p*-value less than 0.01. Red intervals are significantly different from blue intervals.

Serum IL-13 concentrations were significantly lowered in the intervention and washout stages relative to baseline in the stage model (*p* = 0.007, Figure 1). IL-12p70 and fecal lactoferrin were both significantly decreased in the washout stage relative to baseline in the stage model (*p* = 0.01, 0.02). In the week model, only washout stages were lowered in IL-13 (*p* = 0.007).

The overall power of the study was calculated to determine the effectiveness of the current study design. For future studies, we calculated an ideal minimum sample size based on the power of the current study design (Table 5).

## 4. Discussion

Overall, we observed that most cytokines did not change significantly with WPI consumption in our study. However, we detected several significant changes in serum cytokine levels in our population that are potentially attributable to WPI consumption (Figure 1). Serum IL-13 concentrations were significantly lowered in the intervention and washout stages of the stage model relative to baseline. IL-12p70 was significantly decreased in the washout stage of the study relative to the baseline, and fecal lactoferrin was significantly decreased in the washout stage relative to the intervention in the stage model. In the week model, only washout stage weeks 5 and 6 were lowered in IL-13, relative to all other weeks. Many cytokines were detectable only at concentrations below the LOQ. IL-3 was not detected in any of the serum samples.

The NF-κB and MAPK inflammatory pathways were of keen interest, as they have associated cytokines that are easily measured in blood serum and have been implicated in several hypotheses as contributors to chronic inflammation [6,20,21]. They are also thought to be modulated by whey protein consumption and consumption of the whey protein GMP in particular [22]. NF-κB activation is associated with the production of the cytokines IL-1, IL-2, IL-6, IL-8, IL-12 and TNF-α [16]. MAPK-associated cytokines include IFN-γ, IL-1β, IL-1RA, IL-4, IL-6, IL-10 and TNF-α [23,24]. By assessing levels of these cytokines before, during and after whey protein feeding, we attempted to understand if these inflammatory pathways were being modulated by whey protein isolate consumption. In addition to these, other cytokines also associated with inflammation were measured. If these pathways were significantly modulated by whey protein consumption, significant shifts in the expression of several of these markers would be expected; however, among NF-κB- and MAPK-related cytokines, only IL-12p70 was modulated significantly by whey protein consumption. About half of the serum concentrations of Il-12p70 were below the LOQ, and many others were just slightly above, except for participant four. Removing this participant reduced the significance of this difference from *p* = 0.0093 to *p* = 0.0197.

The decrease in IL-12p70 during the washout stage of the study is difficult to interpret, as the effects of whey protein supplementation on IL-12p70 are not well researched. Previous research that involved feeding whey protein concentrate to rats found no change in colonic expression of IL-12p70 [25].

Despite previous research to the contrary [17], we observed no significant changes in IL-6 or other inflammaging-associated cytokines throughout the study. Inflammaging-associated IL-1β and IL-6 concentrations were quite low, with about ⅓ of the data points for both cytokines being below the LOQ. Significant changes were not observed in IL-1β, IL-6 or TNF-α serum concentrations, suggesting that the supplement was ineffective in modulating inflammaging (Figure 1). We observed no significant change in serum IL-6 throughout the duration of the study, an observation that is supported by a recent meta-analysis on the immunomodulatory effects of whey protein intervention [26]. However, whey protein has been shown to lower over-expression of IL-6 in individuals with sarcopenia or frailty [16].

Although they were consistent throughout the study, several serum cytokine concentrations were significantly different from previous multiplex studies on healthy adults, particularly IL-1RA and IL-17 (Table 6). Cytokines were generally detected at concentrations lower than in previous literature. This finding may have been a result of different sample handling/storage conditions, although these are unlikely to explain the large differences in the data observed [27].

Increases in some blood cytokine concentrations are known to occur with age, such as those of IL-2, IL-6, IL-8, IFN-γ and TNF-α [31]. Few studies have broadly assessed serum cytokine levels in healthy adults. However, the serum concentrations of inflammaging-associated IL-1β, IL-6 and TNF-α observed in this study are in line with previous research [21,28].

Serum concentrations of IL-13 were significantly elevated in the baseline stage of the study relative to the intervention and washout. IL-13 is not typically associated with chronic inflammation and is not regulated by the NF-κB and MAPK initial inflammatory responses that modulate most inflammaging-associated cytokines. IL-13 is associated with adaptive immune response rather than innate immune response [32]. IL-13 is also associated with IgE class switching, and its expression has reduced production of inflammatory cytokines in human peripheral blood monocytes exposed to LPS [33,34]. This effect may have been imparted by the GMP component of WPI, as GMP has been observed to significantly decrease IL-13 expression in rodents [35,36,37].

Similarly to IL-13, IL-12p70 is a cytokine that modulates the adaptive immune response, although it can also modulate innate immune signaling [38]. Notably, IL-12 has been observed to down-regulate T-helper (TH)-17 and TH1 signal induction in vitro [38]. This action of IL-12p70 can be considered anti-inflammatory, as TH-17 cells principally produce IL-17 [39], and TH1 cells principally produce IFN-γ [40], both pro-inflammatory cytokines. As such, the decrease in IL-12p70 in the washout stage relative to the baseline may represent a delayed inflammatory bioactivity from WPI consumption.

Beyond IL-13 and IL-12p70, we noticed no significant changes in cytokine concentrations throughout the course of our study. Significant changes would be difficult to observe for most of the markers studied, as they were generally clustered around the limit of quantification for our participants. However, assuming our study participants were healthy, this finding may not be unexpected, as healthy people often have levels of cytokines that are below the limit of detection for commercial analysis kits [31].

Fecal calprotectin and lactoferrin were chosen for investigation because they are clinically important markers of gut inflammation. Though no changes in fecal calprotectin were observed, the observed decrease in fecal lactoferrin in the washout stage relative to the intervention was notable. The baseline stage was found to also be significantly reduced relative to the intervention (*p* = 0.10), suggesting that the intervention may be the stage most modulated by WPI consumption. Indeed, the mean concentrations of fecal lactoferrin were highest in the intervention (Table 3).

However, fecal lactoferrin is not an inherently inflammatory stimulus. Lactoferrin has been found to have anti-inflammatory bioactivities in in vitro models of intestinal epithelial tissue [20]. The decrease in lactoferrin in the washout relative to intervention could be due to effects of whey protein feeding on neutrophil activity. In one study, whey protein extract stimulated the MAPK and NF-κB pathways in human neutrophils as measured by an increase in the expression of inflammatory cytokines in vitro [22]. An increase in neutrophil stimulation can result in increased lactoferrin production in neutrophils at a site for inflammation [41]. This increase could have occurred during the intervention and ended during the washout, explaining the difference.

Our study has several strengths. Most importantly, we chose to examine the effects of WPI consumption on a wide array of serum cytokine concentrations in humans, which had not been completed previously. Analytes were selected to better understand if certain inflammatory pathways could be modulated by WPI consumption. Few studies investigate the effects of whey protein dietary interventions on both blood serum and fecal inflammatory markers, and the significant changes observed in both sample types suggest that it is important to consider both when examining WPI bioactivity.

Our study had some limitations. Our study was limited in size, and it is possible that increasing the number of subjects or samples per week could have allowed additional changes in inflammatory markers to be detected. We computed a power of 35%, which implies that we would detect a small but true effect only 35% of the time (Table 5). Therefore, we may have missed other potential changes in immune marker expression induced by WPI consumption. Based on this power, we determined that a future study of identical design would require 38 participants to achieve a power >80%. Additionally, some multiplex calibration curve data were inconsistent, particularly that for IL-9, for which R^2^ values ranged from 0.624 to 0.904 (Supplementary Table S1). The reason for this finding is not apparent, as all multiplex standards were constructed using the same solution of magnetic beads. However, given that data points remained consistently low and within healthy ranges, it is unlikely that this issue affected data interpretation significantly. With most cytokines investigated being below or only slightly above the limit of quantification, it would be difficult to detect a downregulatory effect of WPI on cytokine expression in healthy adults.

## Figures and Tables

**Table 1:** WPI supplement nutrition facts.

Serving Size	59 g
Calories	230
Nutrients	
Protein	35 g
Carbs	19 g (16 g added sugar)
Fat	0 g
Cholesterol	5 mg
Sodium	220 mg
Calcium	223 mg
Iron	1 mg
Potassium	192 mg

**Table 2:** Descriptive information regarding inflammatory marker assays conducted.

Marker	Range of Quantification (pg/mL) Unless Other Stated	Limit of Detection (pg/mL) Unless Other Stated	Proportion of Values below LOQ
IFN-γ	1.31–20,000	0.86	60/98
IL-1β	1.6–25,000	0.52	31/98
IL-1RA	1.6–25,000	1.29	0/98
IL-2	0.64–10,000	0.28	60/98
IL-3	1.3–20,000	0.28	98/98
IL-4	0.64–10,000	0.2	38/98
IL-5	0.64–10,000	0.17	1/98
IL-6	0.64–10,000	0.14	34/98
IL-7	0.64–10,000	0.14	7/98
IL-8	0.64–10,000	0.52	7/98
IL-9	0.64–10,000	3.05	41/98
IL-10	2.6–40,000	0.91	28/98
IL-12p70	3–50,000	0.88	45/98
IL-13	6.4–100,000	2.58	25/98
IL-17A	1.3–20,000	0.71	42/98
TNF-α	6.4–100,000	5.39	7/98
Fecal calprotectin	4.77–840 [ng/cm^3^]	2.27 [ng/cm^3^]	0/98
Fecal lactoferrin	0.37–240 [ng/cm^3^]	0.37 [ng/cm^3^]	0/98
Total	N/A	N/A	523/1862

**Table 3:** Summary statistics of inflammatory markers in each stage of the trial.

Inflammatory Marker ^1^	Study Stage (Number of Weeks)		
	Baseline, ^2^ (1 Week)	Intervention (3 Weeks)	Washout (3 Weeks)
IFN-γ	5.56 ± 2.77	3.11 ± 0.83	3.79 ± 0.83
Il-1β	8.21 ± 1.92	7.30 ± 1.01	7.79 ± 1.17
IL-1RA	3.48 ± 0.72	3.70 ± 0.49	3.66 ± 0.71
IL-2	1.28 ± 0.39	1.19 ± 0.21	1.01 ± 0.11
IL-4	3.74 ± 2.29	4.12 ± 1.89	3.57 ± 1.48
IL-5	4.39 ± 0.80	3.60 ± 0.33	3.96 ± 0.42
IL-6	1.99 ± 1.01	2.07 ± 0.55	1.62 ± 0.46
IL-7	2.59 ± 0.40	2.97 ± 0.26	2.81 ± 0.29
IL-8	3.23 ± 0.51	3.71 ± 0.38	3.55 ± 0.38
IL-9	32.7 ± 9.8	24.6 ± 4.7	29.8 ± 5.4
IL-10	7.65 ± 1.72	8.46 ± 1.16	7.75 ± 1.12
IL-12p70 ^3^	7.27 ± 2.59 ^A^	5.04 ± 0.76 ^AB^	3.96 ± 0.46 ^B^
IL-13 ^3,4^	52.7 ± 14.3 ^A^	28.9 ± 5.8 ^B^	23.1 ± 4.3 ^B^
IL-17A	5.88 ± 1.73	6.30 ± 1.18	5.65 ± 1.09
TNF-α	24.3 ± 3.70	20.5 ± 2.19	18.9 ± 2.08
Fecal calprotectin	22.2 ± 7.6	31.9 ± 4.7	27.8 ± 4.6
Fecal lactoferrin ^3^	0.09 ± 0.03 ^B^	0.23 ± 0.05 ^A^	0.15 ± 0.02 ^B^

^1^ All IL-3 baseline values were below LOQ. ^2^ Inflammatory marker concentrations are reported as the mean ± standard error (n = 14 for baseline; n = 42 for intervention and washout phases). Concentrations are reported as pg/mL except for fecal calprotectin and fecal lactoferrin, which are reported as μg/mL. ^3^ IL-12p70, IL-13, and fecal lactoferrin concentrations differed significantly by study phase. Inflammatory marker concentrations that do not have shared superscript capital letters following the reported mean ± SEM values indicate statistically significant differences in marker based on study stage (repeated measures ANOVA with Tukey’s post hoc; *p*-value < 0.05). ^4^ IL-13 was the only marker to show a significant difference by week; weeks 5 and 6 were significantly reduced relative to the others (*p*-value = 0.0071).

**Table 4:** Repeated measures ANOVA for inflammatory markers.

Cytokine	Significance in the Stage Model	Significance in the Week Model
IFN-γ	0.20 ^1^	0.47
Il-1β	0.74	0.86
IL-1RA	0.93	0.85
IL-2	0.40	0.22
IL-3	NA	NA
IL-4	0.65	0.70
IL-5	0.11	0.39
IL-6	0.39	0.39
IL-7	0.50	0.64
IL-8	0.51	0.87
IL-9	0.10	0.21
IL-10	0.38	0.79
IL-12p70	0.01	0.07
IL-13	0.0005	0.0071
IL-17A	0.81	0.57
TNF-α	0.28	0.42
Fecal calprotectin	0.41	0.25
Fecal lactoferrin	0.02	0.13

^1^ *p* value for repeated measures ANOVA assessing influence of WPI intervention on serum cytokine, fecal calprotectin and fecal lactoferrin concentrations.

**Table 5:** Study Power Calculation.

Effect Size:	0.2 ^1^
α	0.05
Sample size	14
Number of groups	1
Number of measurements ^2^	3
Correlation among repeated measures ^3^	0.55 ^2^
Computed current study power	0.35
Computed minimum ideal sample size for future studies ^4^	38

^1^ Study power calculated in G*Power using an assumed small effect size of 0.2 [19]. ^2^ Number of groups in ANOVA model (stages in current study). ^3^ Correlation between baseline and intervention inflammatory marker concentrations. ^4^ Determined with power of current study design.

**Table 6:** Comparison of healthy adult serum cytokine concentrations.

Cytokine	This Study	Kleiner et al., 2013 [28]	Kim et al., 2019 [29] ^1^	Biancotto et al., 2013 [30]
IFN-γ	5.51 ^2^	~160	13.1–10.3	543–823 ^3^
Il-1β	8.21	<3.2	2.04–2.52	4.6–4.3
IL-1RA	3.50			279–305
IL-2	1.30	14	5.13–5.18	N/A
IL-3	N/A ^4^	<12		
IL-4	3.70	~8		33.4–36
IL-5	4.40			5.8–8
IL-6	1.98	~11	2.91–2.57	18.3–24.9
IL-7	2.50	13.5		52.2–59.5
IL-8	3.22	29.3	23.9–27.6	38.3–44.2
IL-9	32.7	23.3		113.5–573.3
IL-10	7.65	12.6	3.06–6.16	2.7
IL-12p70	7.04	34.8	7.4–12.2	42.3–53.4
IL-13	52.7	~15		11.2–14.1
IL-17A	5.88			208–202
TNF-α	24.3	~30	3.21–4.94	37–68.7

^1^ Range provided from children to elderly. ^2^ pg/mL. ^3^ Men/Women. ^4^ Undetected.

## Data Availability

The authors can provide all data upon request.

## Supplementary Materials

The following supporting information can be downloaded at: https://www.mdpi.com/article/10.3390/nu15184081/s1, Table S1: Calibration curve R^2^ for each analyte.
