# Supplementary material for: Effects of Whey Protein Supplementation on Inflammatory Marker Concentrations in Older Adults
Source: Nutrients. 2023 Sep 21;15(18):4081. doi: 10.3390/nu15184081 (PMC10534557; doi:10.3390/nu15184081)
Supplement: Supplementary file 1 [file nutrients-15-04081-s001.zip › nutrients-2554698-supplementary.pdf]

Table S1: Calibration curve R^2 for each analyte

Marker	Run 1 (Participants A-E)	Run 2 (Participants F-J)	Run 3 (Participants K-N)
IFN-γ	0.937	0.968	0.843
IL-1β	0.965	0.982	0.887
IL-1RA	0.977	0.999	0.980
IL-2	0.925	0.987	0.957
IL-3	0.900	0.996	0.996
IL-4	0.890	0.970	0.995
IL-5	0.847	0.992	0.959
IL-6	0.965	0.993	0.894
IL-7	0.889	0.99	0.982
IL-8	0.927	0.994	0.980
IL-9	0.624	0.904	0.649
IL-10	0.935	0.995	0.971
IL-12p70	0.936	0.953	0.984
IL-13	0.919	0.980	0.821
IL-17A	0.936	0.987	0.981
TNF-α	0.945	0.987	0.989
Fecal calprotectin	0.997	0.994	0.988
Fecal lactoferrin	0.940	0.963	0.981
